# Stepwise Localization of Non‐Pulmonary‐Vein Ectopy Using Cycle‐Length‐Based Activation Map Separation and Local Electrogram Morphology on the Affera System

**DOI:** 10.1002/joa3.70484

**Published:** 2026-09-27

**Authors:** Suguru Nishiuchi, Shingo Yoshimura, Takehito Sasaki, Kohki Nakamura, Shigeto Naito

**Affiliations:** ^1^ Division of Cardiology Gunma Prefectural Cardiovascular Center Maebashi Gunma Japan

**Keywords:** activation mapping, Affera, cycle length, premature atrial contraction, Sphere‐9, superior vena cava, voltage‐per‐second

## Abstract

On the Affera system, a single voltage‐map dataset containing both sinus and ectopic beats, collected without rhythm‐specific settings, can be separated by cycle length (coupling interval) into SR and PAC activation maps. Stepwise filtering by activation threshold (voltage‐per‐second, excluding far‐field potentials) and close‐unipolar QS‐like morphology then precisely localizes the ectopic origin.

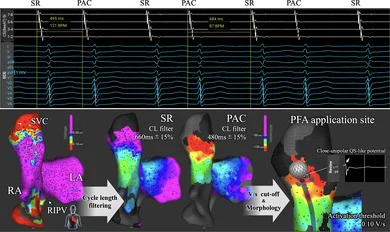

Ectopic atrial activity arising outside the pulmonary veins (PVs), including from the superior vena cava (SVC), is a recognized trigger of atrial fibrillation (AF) [1, 2], but such ectopy is often infrequent or provoked only transiently, making it difficult for conventional, rhythm‐restricted mapping workflows to localize. Existing solutions, including self‐reference mapping [3] and pace‐mapping strategies such as the Intracardiac Pace Match Scoring (iPASO) technique, require a dedicated mapping or pacing step built specifically around the ectopic beat [4].

The Affera Prism‐2 mapping system (Medtronic) acquires voltage‐map points using a fixed collection window referenced to a chosen channel, without any cycle‐length (CL) or pattern‐matching restriction applied at the time of acquisition. Every point within the window is accepted regardless of the underlying rhythm, so sinus and ectopic beats can be recorded together within a single voltage‐map point dataset. Rhythm discrimination is deferred to a later step: when an activation map is generated, each point's preceding CL can be used as a retrospective filter, so the same mixed‐rhythm point dataset yields separate, co‐registered activation maps for each rhythm. In addition, the system's voltage‐per‐second (V/s) filter, labeled “activation threshold” in the activation‐mapping setting and representing the maximum local slew rate, can be raised to progressively restrict the displayed points to those with the steepest, most near‐field‐dominant signals [5]. Raising this threshold helps exclude far‐field potentials and reduces the risk of misannotating inaccurate signals as local activation.

In the representative case, a 75‐year‐old man with a 10‐year history of paroxysmal AF, previously treated with pulmonary vein isolation (PVI) and cavotricuspid isthmus ablation, underwent redo ablation for recurrent AF. After confirming durable PVI and completing left atrial posterior wall isolation, an intravenous isoproterenol bolus (4 μg) induced an episode of incessant AF, followed by frequent premature atrial contractions (PACs), with a coupling interval of 410–510 ms superimposed on an underlying sinus CL of 610–700 ms (Figure 1, panel A). The atrial activation sequence of these PACs was similar to that of the beat that had initiated the AF episode, suggesting a shared origin. However, because this PAC did not reproducibly induce AF on every occurrence, we refer to it as non‐pulmonary‐vein ectopy, a probable AF trigger. Post‐procedural voltage mapping of the left atrium, PVs, right atrium, and SVC was partially performed using the Sphere‐9 catheter. Because acquisition under the Affera voltage‐mapping protocol was unrestricted, SR and PAC points were collected together within a single voltage mapping point dataset (Figure 1, panel B). This point dataset was separated during the procedure, using the CL filter, into an SR‐CL map (660 ms) and a PAC‐CL map (480 ms), with a ±20% margin of tolerance applied to identify the origin. Because this tolerance window overlapped mathematically between the two filters, the maps were retrospectively reconstructed using a narrower ±15% tolerance (561–759 and 408–552 ms, respectively), which eliminated the overlap; the resulting activation pattern was equivalent to that obtained with the original setting. Few beats fell within the overlapping range in this case, but this potential overlap should be considered when selecting the tolerance setting. The PAC‐CL map showed the earliest activation cranial to the sinoatrial node, extending into the SVC (Figure 1, panels C and D).

**FIGURE 1:**
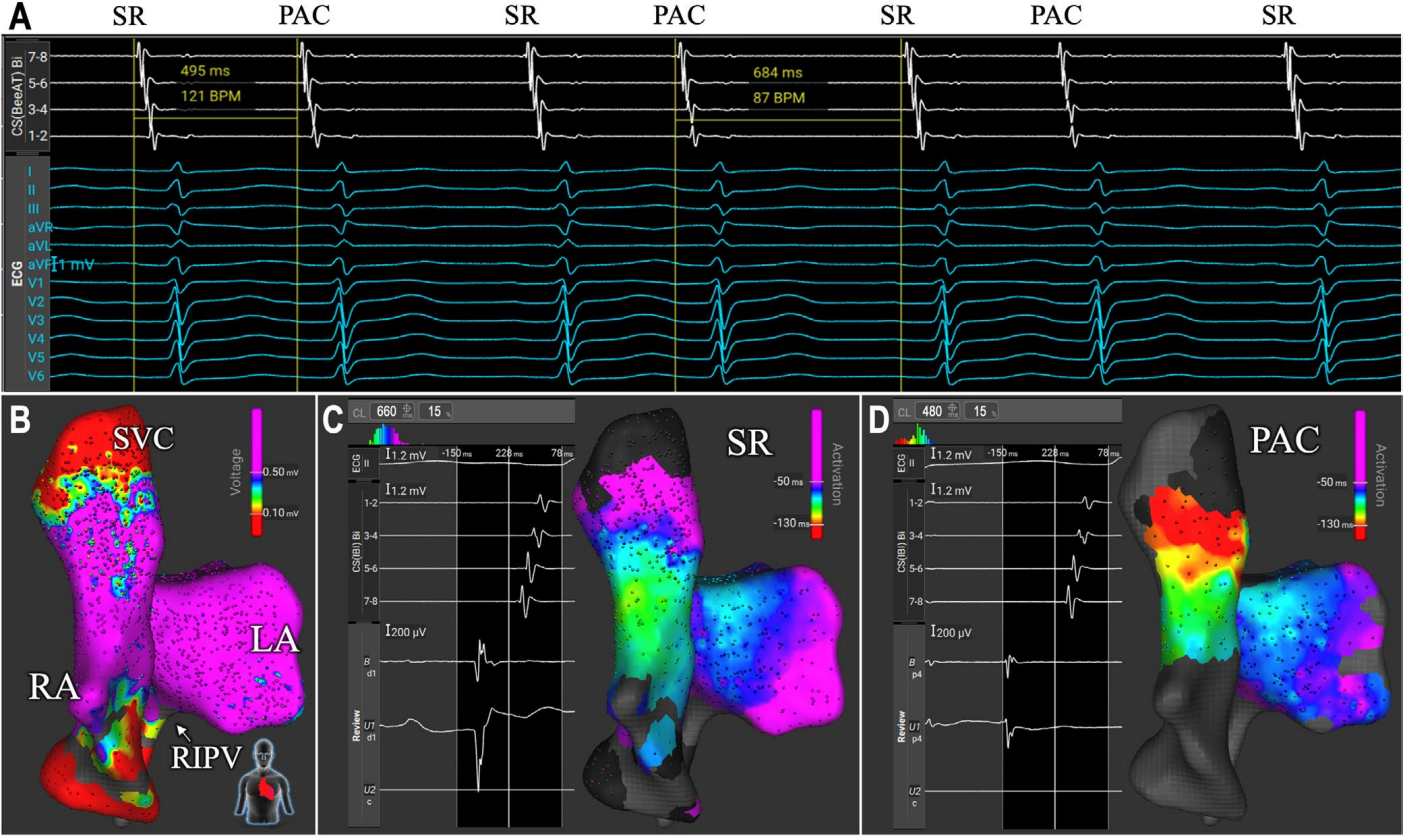
Cycle‐length‐based separation of sinus and PAC activation maps. (A) Coronary sinus bipolar electrograms and body surface 12‐lead ECG showing SR beats and the isoproterenol‐provoked PAC. (B) Partial voltage maps of the left atrium, right atrium, and SVC, acquired with the CS catheter as the window reference (RIPV = right inferior pulmonary vein, labeled for anatomical orientation). (C) SR‐CL activation map (660 ms), showing earliest activation near the SN region. (D) PAC‐CL activation map (480 ms), showing earliest activation cranial to the SN region, extending into the SVC. CL, cycle length; CS, coronary sinus; ECG, electrocardiogram; PAC, premature atrial contraction; RIPV, right inferior pulmonary vein; SN, sinoatrial node; SR, sinus rhythm; SVC, superior vena cava.

To identify the precise target within this region, the V/s filter was progressively raised from 0.02 to 0.06 and then to 0.10 (Figure 2), narrowing the earliest‐activation area to a small cluster of points at each step; these threshold values were selected empirically for this case rather than representing validated, universal cutoffs for focal atrial ectopy. A high V/s cut‐off value helped exclude the misannotation of far‐field potentials or inappropriate signals (Figure 2, panels A and B). Within the early‐phase annotated points with the high V/s setting, the individual close‐unipolar electrograms, recorded using the Sphere‐9 catheter's central indifferent electrode configuration, were manually reviewed to identify the site with the smallest‐amplitude initial positive deflection, closest to a QS pattern, a feature generally associated with closer proximity to the site of origin (Figure 2, panels B–D). Pulsed field ablation at this site suppressed the PAC. An additional application was performed, but there was no phrenic nerve palsy. The patient remained arrhythmia‐free at the 6‐month follow‐up.

**FIGURE 2:**
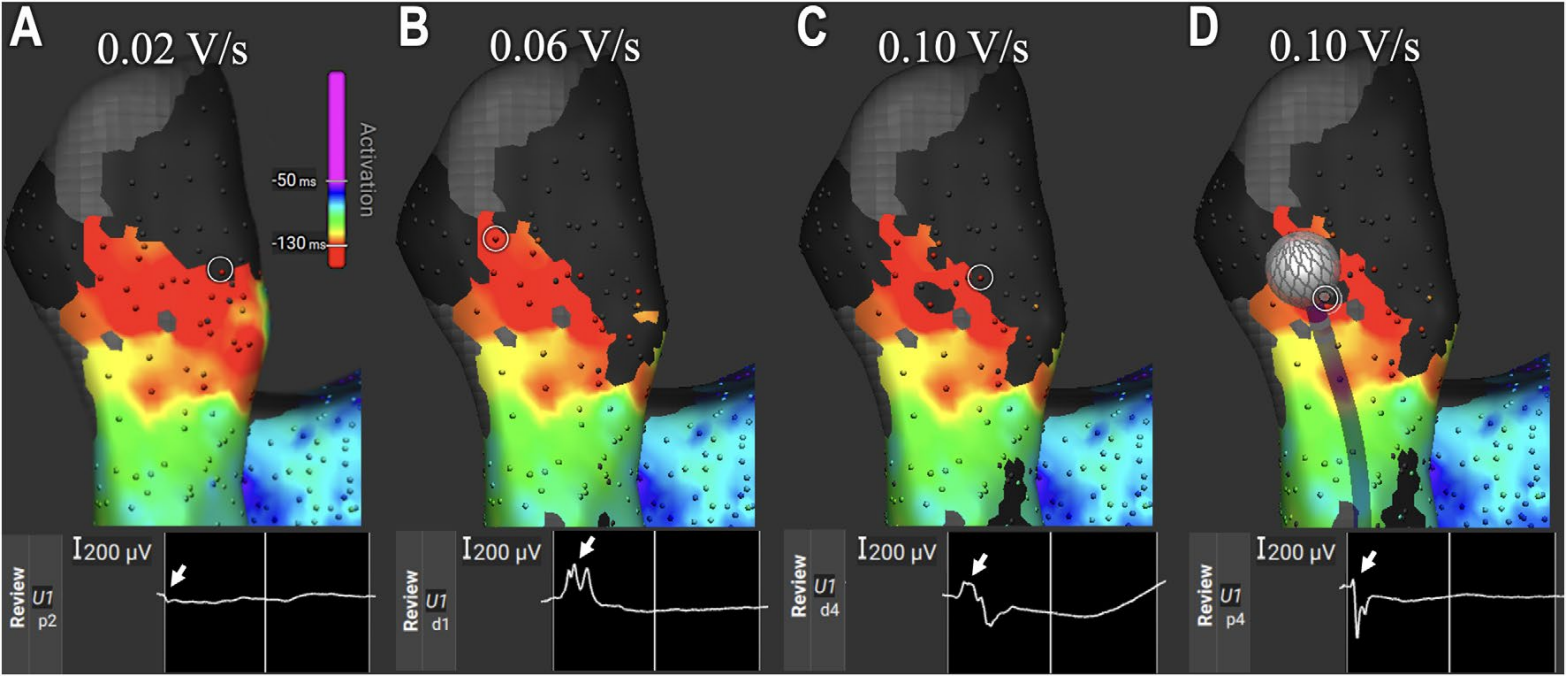
Stepwise narrowing of the ectopy localization using the voltage‐per‐second (V/s) filter (activation threshold) and local close‐unipolar potential of the encircled mapping point in each map. These V/s thresholds were selected empirically for this case. (A) V/s ≥ 0.02, the default activation‐map setting; accurate localization of the ectopic site is difficult, and a dull potential is annotated in the early‐activation phase. (B) V/s ≥ 0.06; this relatively higher cutoff excludes the dull potential seen in panel A. (C) V/s ≥ 0.10, showing further narrowing of the candidate area; however, the local close‐unipolar potential still includes an initial R wave in the early‐activation region (local potentials in panels B and C). (D) Close‐unipolar electrogram at the finally selected site, showing a QS‐like morphology with a small initial r deflection; this QS‐like morphology, rather than the residual R wave seen in panel C, supported closer proximity to the focal origin. The catheter indicates the successful pulsed field ablation application point.

We have since applied this stepwise workflow, consisting of CL‐based map separation, high V/s filtering, and manual close‐unipolar morphology determination, in three additional cases of provoked non‐pulmonary‐vein ectopy (one at the right atrium–inferior vena cava junction, two in the left atrium). The eventual successful ablation site was a median of 7 mm from the site identified by this approach in all four cases. Further research and validation of this approach in a larger cohort will be required.

The present mapping process required no dedicated re‐mapping step beyond the ongoing map already being performed for another purpose, such as the voltage map acquired after PFA application, because Affera's voltage map does not gate acquisition by rhythm during the procedure. This stepwise approach may represent a novel addition to the Affera mapping workflow for localizing transient non‐pulmonary‐vein ectopy captured incidentally during routine mapping.

## Funding

The authors have nothing to report.

## Conflicts of Interest

The authors declare no conflicts of interest.

## Data Availability

The data that support the findings of this case report are available from the corresponding author upon reasonable request.
